# Delayed presentation of penile strangulation by a foreign object: Case report with review of literature

**DOI:** 10.1016/j.ijscr.2025.112050

**Published:** 2025-10-13

**Authors:** Manzoor Ahmad dr, Ezaz Ahmed, Ahmad Sadiq, Wasif Mohammad Ali

**Affiliations:** Department of Surgery, JNMCH, AMU, Aligarh, 202002, Uttar Pradesh, India

**Keywords:** Penile strangulation, Metal ring, Urological emergency, Aspiration technique, String method, Case report

## Abstract

**Introduction and importance:**

Penile strangulation is a rare urological emergency first described in 1755, often resulting from foreign object entrapment leading to vascular compromise, oedema, and potentially necrosis or gangrene. It is associated with delayed presentation due to patient embarrassment and lacks standardized management protocols. This report emphasizes the clinical challenges and outcomes associated with delayed presentation and highlights a successful conservative management approach.

**Case presentation:**

A 24-year-old unmarried South-Asian male with a history of cannabis use and abnormal behaviour presented with penile strangulation by a thick metallic ring of 96 h duration. Examination revealed Grade II penile injury with oedema and distal congestion. Initial decompression attempts failed. Penile block followed by aspiration of corpora cavernosa and use of feeding tubes alongside lubrication allowed successful removal via the string method. The patient developed a localized superficial infection managed with intravenous Piperacillin–Tazobactam following *Escherichia coli* growth in wound swab culture. He responded well and was discharged on postoperative day 7.

**Clinical discussion:**

Penile strangulation requires individualized treatment based on severity. Minimally invasive techniques like aspiration and string method are effective for low-grade injuries. Literature supports emerging techniques involving non-traditional tools such as dental drills and air cutters, particularly in delayed or complex presentations. Complications like infection and necrosis are more common with delayed presentation, emphasizing the need for early intervention and public awareness.

**Conclusion:**

Early recognition, appropriate technique selection, and multidisciplinary care are key to preventing long-term sequelae. This case reinforces the effectiveness of conservative decompression methods in low-grade injuries despite delayed presentation.

## Highlights

• Delayed presentation of penile strangulation is rare as most patients present early
• Management of the wound including antibiotic administration and regular dressing
• Infection of the wound is also an uncommon finding in such cases

## 1 Introduction

Penile strangulation is a rare urological emergency that was first reported by Gautier in 1755 [1]. Penile entrapment by an object causes blockage of its vascular and lymphatic channels resulting in mild oedema, necrosis, sepsis or gangrene depending on the time of presentation to the hospital and subsequent removal of the object [2,3]. Although the incidence of this condition is unknown, only a few case series and reports have been published till date [4]. Penile rings are utilized to increase penoscrotal engorgement or may be used as sexual toys and may be used by individuals with erectile dysfunction or psychosexual disorders to enhance sexual gratification, thus sexual behavioural knowledge should be widely spread, especially to the younger population [5].

According to the existing medical literature, management of this condition is challenging as there is no standard guideline owing to the heterogenous nature of presentation and its associated complications. Generally they are managed on a case-to-case basis [6].

Herein, we report a case of penile strangulation by a round metal ring which was resolved by the aspiration and string method. The subsequent course of the disease was further complicated by an associated infection of the superficial skin.

## 2 Case presentation

**Fig. 1:**
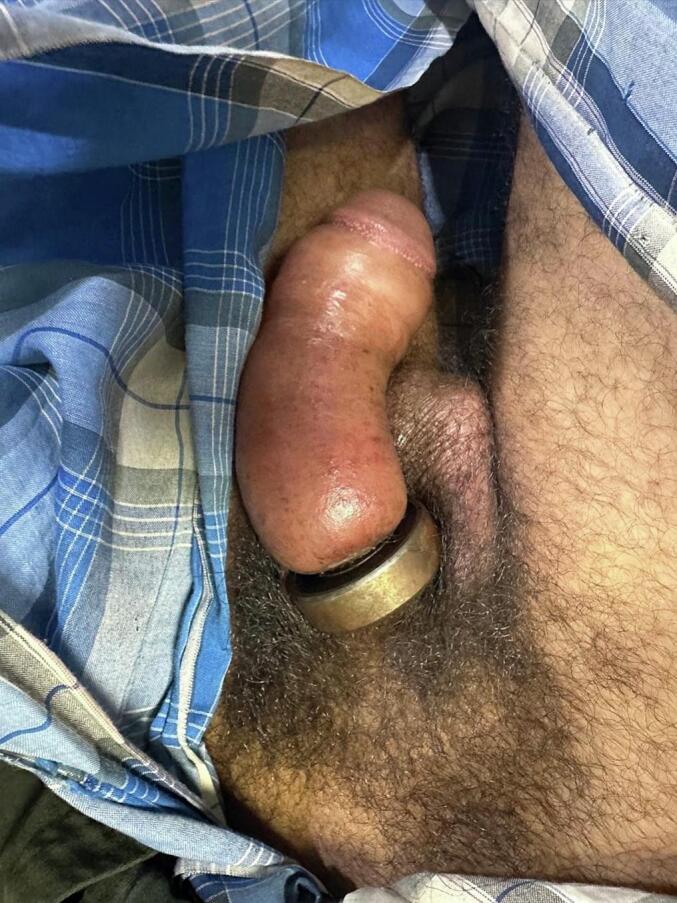
Image showing a steel ring at the base of the penis with oedema of the distal penile shaft.

A 24 year old unmarried South-Asian male presented to the emergency room with penile strangulation for 96 h duration by a thick metal ring. He had a past history of irrelevant talk, irritability and anger outburst associated with a history of cannabis abuse and cigarette smoking for the last 6–7 years. He complained of pain and enlargement of the penile shaft. His vitals were stable at the time of presentation. The patient was irritable with disorganised behaviour. The patient was circumcised and the metal ring which measured 2 cm in diameter was located at the base of the penis. Local examination demonstrated that the temperature of the penile shaft was decreased, the shaft distal to the constricting metal object was tender, oedematous and congested with intact distal sensations. There were no signs of necrosis or gangrene in the glans or the shaft of the penis [Fig. 1]. This case is typically a Grade II penile injury according to the Bhat Classification and low grade injury according to the Silberstein classification [3,7].

Following initial evaluation, he was resuscitated with IV fluids and antibiotics. As the patient was not in urinary retention at presentation so urinary catheterisation was not attempted. Initial attempts at manual decompression using lubricant to remove the metal ring were not successful due to the existing oedema.

**Fig. 2:**
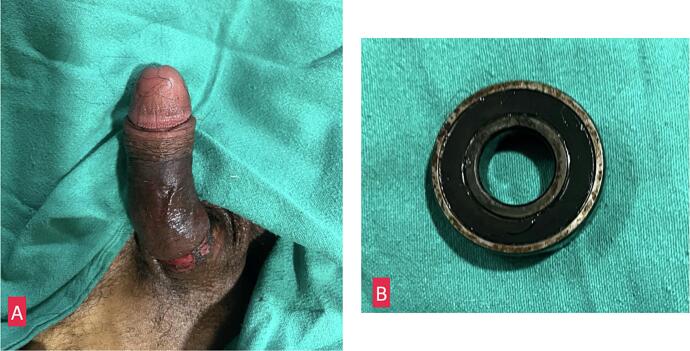
A: Image showing immediate post procedure condition of the penile shaft with mark of constriction where the ring was lodged B: Thick metallic ring that was removed.

After penile block, two wide bore needles (16 gauge) were inserted into the distal corpora cavernosa and blood aspirated. After that, manual compression of the distal penis led to partial decompression of the penis. After proper lubrication with 2 % lignocaine jelly, two 10 Fr feeding tubes could be negotiated between the ring and the penis from opposite ends and the ring was sided from the root of the penis towards the glans. The procedure was repeated and the ring was removed (Fig. 2 a/b). The patient was also seen by the psychiatrist and was started on Sodium Valproate and a combination of Fluoxetine and Olanzapine for symptomatic relief of his abnormal behavioural.

**Fig. 3:**
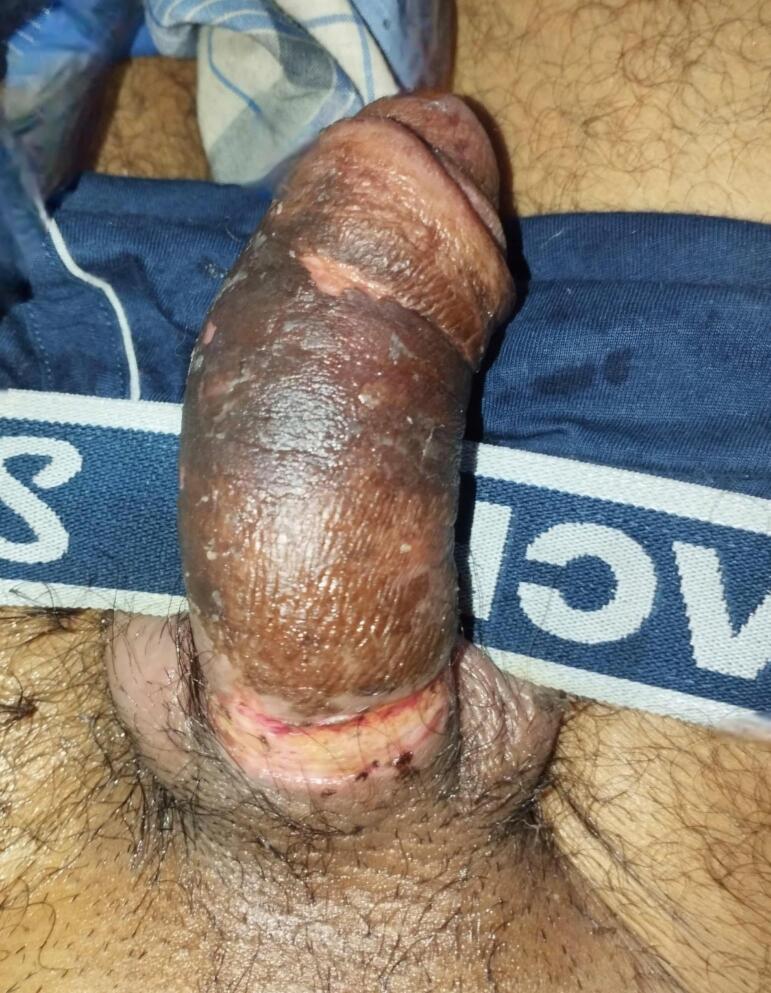
Image showing the condition of the penis three days post procedure with presence of pus at the site of constriction by the ring.

**Fig. 4:**
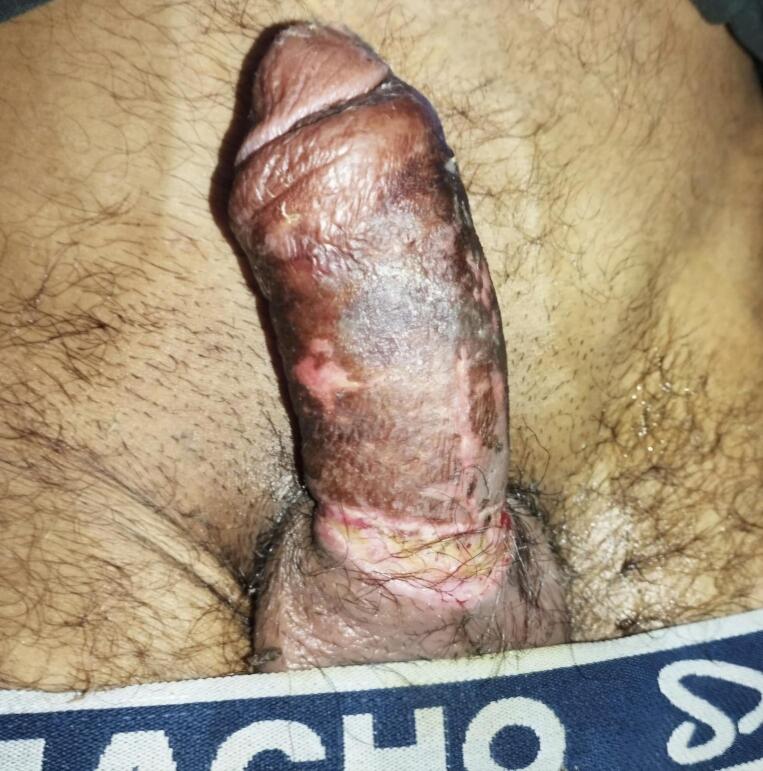
Image showing the condition of the penis at the time of discharge with depigmentation of overlying skin.

The patient developed a constriction ring mark at the site of the metal ring in the post procedural period (Fig. 3). Daily dressing of the wound was done with normal saline but subsequently pus was seen around the wound on post operative day 2. His total leucocyte count was raised to 13,200/mm^3^. Swab culture was sent which showed growth of *Escherichia coli*, which was sensitive to Pipercillin-Tazobactum and Meropenem. The patient was started on Injection Pipercillin-Tazobactum 4.5 g 8 hourly and daily dressing was continued. Pus discharge from the wound decreased and the patient was discharged on post procedure day 7 (Fig. 4). Tenth day follow up showed healed wound with a mark of constriction ring around the base of the penis. The patient was voiding normally and there was no loss of sensation of the distal penis. Long term follow up could not be done as the patient refused to turn up.

## 3 Discussion

Penile strangulation, while rare, represents a urological emergency which necessitates prompt and tailored intervention. A comprehensive review of 100 cases from 2000 to 2019 reported long-term sequelae in 24 % of patients, including urethral strictures, fistulae, and sensory loss—though 76 % achieved full but delayed recovery when managed early [8]. This highlights the critical importance of both timely removal and prolonged follow-up care in mitigating complications.

Innovative removal techniques are emerging. Nguyen et al. (2022) described using a dental diamond drill handpiece to cut a thick steel nut after 10 h of incarceration. With continuous cooling and tissue shielding, the nut was removed without skin injury, and no urinary or erectile complications were observed [9]. Similarly, a 2024 case utilized a high-speed air cutter by an emergency rescue team, successfully releasing a constricting ring in 90 min while protecting the penile skin [10]. These non-traditional tools, paired with protective measures, offer effective alternatives when standard equipment fails. Traditional cutting tools remain crucial, especially for metallic objects. A 2021 review documented multiple cases employing Dremel moto-tools, orthopedic saws, bolt cutters, angle grinders, and dental drills—often necessitating General Anesthesia or conscious sedation. The choice depends on device type, size, and patient stability [10].

Delayed presentation—frequently due to embarrassment—complicates outcomes. For example, Singh et al. (2022) reported a case of a metallic ring trapped for 4 days, causing necrosis and infection that required surgical debridement [11]. Not a big role of detumuscence exist in such cases, because the underlying pathology is oedema and swelling of the penile shaft. This underscores the need for public education and clinical vigilance. In our case, the patient developed superficial skin infection for which IV antibiotic was given that resulted in prolonged hospital stay.

Management algorithms stratify interventions based on injury grade. Low grades (I–III) often respond to aspiration, string technique, or simple cutting. Higher grades (IV–V), which involve deep tissue injury, typically require advanced tools or surgical degloving. Each step must consider thermal protection, tissue preservation, and anesthesia planning. Imaging like a Doppler Ultrasonography of the penile shaft may aid in further evaluation of any tear in the tunica albugenia which may warrant a surgical repair. Current literature emphasize three key pillars, namely early presentation and classification, appropriate, innovative tools and comprehensive follow-up.

Our case aligns with this model: using minimally invasive method of removal, managing subsequent infection, and recommending long-term surveillance. As newer equipment becomes more accessible, emergency departments and urology teams should update protocols to include these options and ensure safe application. This case reinforces the need for multidisciplinary care—including urological, microbiological, and psychiatric input—to optimize outcomes and reduce the risk of irreversible damage.

## 4 Conclusion

Penile strangulation is an uncommon urological emergency that demands timely and individualized intervention to prevent irreversible vascular and tissue damage. Our case highlights the successful use of the aspiration and string method for removal of a metallic constricting object in a young male with a history of substance abuse. Despite delayed presentation, prompt decompression, vigilant postoperative care and IV antibiotics prevented major sequelae.

## Consent

Written consent was taken from the patient and may be obtained as per requirement.

## Ethical approval

Ethical approval was exempted from the ethical committee.

## Guarantor

I, Ezaz Ahmed, the corresponding author of the case, accept full responsibility for the work and/or conduct of the study.

## Funding

No funding procured.

## Research registration

Not applicable.

## Author contribution

Manzoor Ahmad: Study concept and design, Supervision.

Ezaz Ahmed: Writing of paper.

Ahmad Sadiq: Supervision.

Wasif M Ali: Supervision.

## Declaration of competing interest

The authors have declared that no competing interests exist. This case report has been reported in line with the SCARE checklist [12].
